# Stagnation of maternal mortality decline in Bangladesh between 2010 and 2016 in spite of an increase in health services utilisation: Examining data from three large cross-sectional surveys

**DOI:** 10.7189/jogh.14.04027

**Published:** 2024-01-26

**Authors:** Karar Zunaid Ahsan, Gustavo Angeles, Siân L Curtis, Peter Kim Streatfield, Nitai Chakraborty, Mizanur Rahman, Kanta Jamil

**Affiliations:** 1 Department of Public Health Leadership and Practice, University of North Carolina at Chapel Hill, Chapel Hill, North Carolina, USA; 2 Data for Impact (D4I) and Department of Maternal and Child Health, University of North Carolina at Chapel Hill, Chapel Hill, North Carolina, USA; 3 Health Systems and Population Studies Division (HSPSD), International Centre for Diarrhoeal Disease Research, Dhaka, Bangladesh; 4 D4I, University of North Carolina at Chapel Hill, Chapel Hill, North Carolina, USA; 5 Independent public health researcher, Melbourne, Australia

## Abstract

**Background:**

After a 40% reduction in maternal mortality ratio (MMR) during 2001–2010 in Bangladesh, the MMR level stagnated between 2010 and 2016 despite a steady increase in maternal health services use and improvements in overall socioeconomic status. We revisited the factors that contributed to MMR decline during 2001–2010 and examined the changes in these factors between 2010 and 2016 to explain the MMR stagnation in Bangladesh.

**Methods:**

We used data from the 2001, 2010, and 2016 Bangladesh Maternal Mortality Surveys, which sampled 566 115 households in total, to estimate the changes in the risk of dying of maternal causes associated with a pregnancy or birth between 2001–2010 and 2010–2016. We carried out Poisson regression analyses with random effects at the sub-district level to explore the relationship between the change in risk of maternal death from 2001 to 2016 and a range of demographic, socioeconomic, and health care factors.

**Results:**

Between 2001 and 2016, the proportion of high-risk pregnancies decreased, except for teenage pregnancies. Meanwhile, there were notable improvements in socioeconomic status, access to health services, and the utilisation of maternal health services. A comparison of factors affecting the risk of maternal death between 2001–2010 and 2010–2016 indicated that first pregnancies continued to offer significant protection against maternal deaths. However, subsequent pregnancies among girls under 20 years became a significant risk factor during 2010–2016, increasing the risk of maternal deaths by nearly 3-fold. Among the key maternal health services, only skilled birth attendants (SBA) were identified as a key contributor to MMR reduction during 2001–2010. However, SBA is no longer significantly associated with reducing mortality risk during 2010–2016.

**Conclusions:**

Despite continued improvements in the overall socioeconomic status and access to maternal health services in Bangladesh, the stagnation of MMR decline between 2010 and 2016 is associated with multiple teenage pregnancies and the lack of capacity in health facilities to provide quality delivery services, as SBA has been primarily driven by facility delivery. The findings provide a strong rationale for targeting at-risk mothers and strengthening reproductive health services, including family planning, to further reduce maternal mortality in Bangladesh.

Globally, nearly 300 000 mothers die from preventable causes related to pregnancy and childbirth, of which 94% occur in low-income and lower-middle-income countries [1]. Improving maternal health by increasing coverage of reproductive health services and reducing maternal morbidity, as well as mortality, has long been a priority within the global development agenda. Indicators of maternal mortality and coverage of essential maternal health services have been a part of the United Nations Millennium Development Goals (MDGs) for 2015 and the Sustainable Development Goals (SDGs) for 2030 [2,3]. The Government of Bangladesh is committed to improving the coverage of critical maternal and reproductive health services in the country and reducing the maternal mortality ratio (MMR) to 105 deaths per 100 000 live births in 2022 [4]. Through a series of sector-wide approaches since 1998, the government and development partners have been investing in Bangladesh’s health sector to increase the access to and utilisation of essential reproductive health services in Bangladesh.

Bangladesh is the only low- or lower-middle-income country with three high-quality, nationally representative household sample surveys measuring maternal mortality and health services use. The 2001, 2010, and 2016 rounds of the Bangladesh Maternal Mortality and Health Care Survey (BMMS) were conducted by the National Institute of Population Research and Training (NIPORT) under the auspices of the Ministry of Health and Family Welfare. The surveys aimed to estimate maternal mortality in Bangladesh by collecting data on specific causes of death and women's experiences with maternal care. Funding was provided by a combination of the government and development partners: the United States Agency for International Development (2001, 2010, 2016); the Government of Bangladesh (2010, 2016); the Australian Agency for International Development (2010); the United Nations Population Fund (2010); and the United Kingdom's Department for International Development (2016). Technical assistance for these surveys was provided by the United States Agency for International Development via the Demographic and Health Surveys Program (DHS) (2001); Johns Hopkins University (2001); the International Centre for Diarrheal Disease Research, Bangladesh (2001, 2010, 2016); and the MEASURE Evaluation project (2010, 2016). Data from BMMS rounds demonstrated that MMR in Bangladesh declined from 322 in 2001 to 194 per 100 000 live births in 2010, but there is no evidence of a further reduction in MMR after 2010 [5]. Despite continued improvement in a number of maternal and reproductive health care utilisation indicators [6], the estimated maternal mortality ratio (MMR) in BMMS 2016 was 196 per 100 000 live births [5]. Given the government and the development partners’ commitment to reducing maternal mortality in Bangladesh, no evidence of a reduction in MMR between the 2010 and 2016 BMMSs warrants a thorough examination of factors affecting maternal mortality.

In an earlier study, we explored how Bangladesh achieved a 40% reduction in maternal mortality using the 2001 and 2010 rounds of BMMS. We found that the decline in MMR in Bangladesh seemed to have resulted from factors within and outside the health sector [7]. In this paper, we revisited the factors that contributed to the reduction in MMR during 2001–2010 and examined the changes in these factors between 2010 and 2016 to explain the stagnation in MMR in Bangladesh. Based on the review of Bangladesh’s progress in maternal health and international experiences in reducing maternal mortality, we outlined several policies as well as programmatic recommendations to reduce MMR in the coming years.

## METHODS

### Data sources

We used data from the 2001, 2010, and 2016 rounds of BMMS for this study. The BMMSs were designed to assess the country’s maternal health situation and provide national MMR estimates. These surveys were extensive and nationally representative – using a three-stage sampling design, BMMS covered 99 202 households in 2001, 168 629 households in 2010, and 298 284 households in 2016. Details of the survey design can be found elsewhere [5,8,9]. As stated earlier, these three rounds of BMMS are the only data set available for Bangladesh that allows us to explore the association between the risk of maternal death and relevant demographic, socioeconomic, and health care factors using household-level data with acceptable precision. In order to explain the stalling of maternal mortality decline during 2010–2016, we first compared the MMR trajectory estimated from BMMS with other in-country data sources (Supplement 1 in the **Online Supplementary Document**) and re-examined the factors behind the MMR reduction during 2001–2010 using these three rounds of survey data.

Deaths of women of reproductive age, occurring approximately four years prior to the surveys, were followed up for verbal autopsy using an adapted version of the WHO structured questionnaire and classified according to the International Classification of Diseases, 10th Revision [10,11]. Data from verbal autopsies were reviewed independently by two separate physicians to assign the cause of death, inclusive of maternal causes. In cases of disagreement on the cause of death between the two initial reviewers, a third physician reviewed the data to determine the cause of death. In addition to verbal autopsy data, all three cycles of the BMMS gathered information on selected background characteristics, complete birth history from surviving ever-married women aged 13–49 years, and several maternal health indicators. These indicators included the number of issues experienced during the recent pregnancy and delivery, health-related actions taken, and the extent of service coverage for selected maternal health care services [5,8,9].

### Statistical analysis

We pooled data of live births from surviving women and pregnancies from women who died from maternal causes within the three years prior to each survey. This was done utilising the data from three rounds of the BMMS. The pooled data set included variables on the mother’s death due to maternal causes, her socio-demographic characteristics, and her behaviours related to seeking maternal care. We computed service use indicators as sub-district level averages for surviving women to address the concerns about reverse causation between service utilisation and obstetric risk. We primarily used the same definition for the variables from our earlier study so that we could examine the changes in these factors between 2001–2010 and 2010–2016 to explain the stagnation in MMR in Bangladesh [7].

To examine the factors associated with the change in maternal mortality between 2001–2010 and 2010–2016, we carried out a Poisson regression analysis with random effects at the sub-district level on the pooled data set. Poisson model at the individual pregnancy/birth level was set up following the regression specification shown below to explore relationships between the risk of dying of maternal causes associated with a pregnancy or birth and the selected factors:



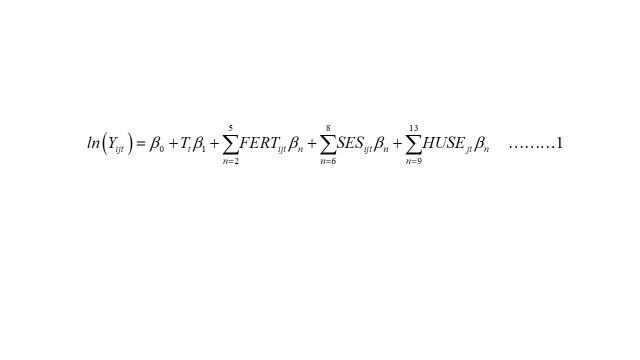



Where *Y_ijt_* is a dummy variable indicating whether the pregnancy *i* living in sub-district *j* in the three years before survey *t* resulted in a maternal death; *T_t_* is a dummy variable for survey rounds (for 2001–2010, 2001 = 0 and 2010 = 1; for 2010–2016, 2010 = 0 and 2016 = 1); *FERT_ijt_* is a vector of four explanatory demographic variables (i.e. fertility-related risk factors for maternal death such as first pregnancy, fourth or higher pregnancy, age at birth under 20, age at birth over 35) for mother *i* at survey *t*; SE*S_ijt_* is a vector of three explanatory socioeconomic variables: the place of residence (rural or urban), household wealth quintiles, and years of education for mother *i* at survey *t*; and *HUSE_jt_* is a vector of three explanatory service utilisation variables (i.e. the proportion of births delivered by caesarian section, proportion of deliveries with at least four antenatal care visits, and proportion of deliveries by a skilled birth attendant (SBA, which includes qualified doctor, nurse/midwife/paramedic, family welfare visitor, community skilled birth attendant, and medical assistant/sub-assistant community medical officer) in sub-district *j* at survey *t*. Bivariate analysis and analyses using the *xtpoisson* routine were used for all primary regressions with random effects at the sub-district level in the Stata statistical software, version 17, to estimate the association between the risk of maternal death and various factors during 2001–2010 and 2010–2016 [12].

## RESULTS

### Sample characteristics

**Table 1** provides the proportions of births/pregnancies by variables of interest. Among the 566 115 sampled households over the survey rounds, 216 525 ever-married women of reproductive age were selected for analyses who were pregnant or had childbirth in the three years preceding each BMMS round. Data showed that maternal mortality among the sampled respondents significantly decreased between 2001 and 2010, from 342 deaths to 213 deaths per 100 000 pregnancies (*P* < 0.05). However, between 2010 and 2016, maternal mortality decreased only from 213 deaths to 186 deaths per 100 000 pregnancies, and this reduction was not statistically significant. This is to be noted that for this statistical analysis, we decided to focus on obstetric risk, or risk per pregnancy, as measured by MMR. The MMR estimates shown in **Table 1** are not exactly the same as the conventionally estimated MMR [5] (Supplement 1 in the **Online Supplementary Document**).

**Table 1:** Descriptive statistics by selected variables for 2001, 2010, and 2016 BMMS

Sample characteristics	Mean (95% confidence interval)		
**2001**	**2010**	**2016**
**Outcome variable**			
Maternal death	0.00342 (0.00293–0.00392)	0.00213 (0.00177–0.00250)	0.00186 (0.00160–0.00213)
**Demographic variables**			
First pregnancy	0.238 (0.235–0.242)	0.325 (0.321–0.328)	0.389 (0.386–0.392)
Birth parity ≥4	0.358 (0.354–0.362)	0.214 (0.211–0.217)	0.126 (0.124–0.128)
Birth at age <20 y	0.191 (0.188–0.194)	0.151 (0.148–0.154)	0.284 (0.282–0.287)
Birth at age ≥35 y	0.114 (0.111–0.116)	0.088 (0.086–0.090)	0.046 (0.045–0.047)
**Socioeconomic variables**			
Years of education	3.108 (3.078–3.139)	4.994 (4.964–5.023)	6.634 (6.610–6.657)
Economic status	2.776 (2.764–2.788)	2.912 (2.900–2.923)	2.968 (2.959–2.977)
Urban residence	0.173 (0.170–0.176)	0.237 (0.234–0.240)	0.263 (0.261–0.266)
Maternal care-seeking variables*			
≥4 antenatal visits	0.112 (0.111–0.113)	0.222 (0.221–0.223)	0.356 (0.355–0.357)
Skilled delivery attendants	0.116 (0.115–0.117)	0.266 (0.265–0.267)	0.499 (0.498–0.500)
Cesarean sections	0.026 (0.026–0.026)	0.123 (0.122–0.124)	0.308 (0.307–0.309)
**Access to health services variables†**			
Public health facility <1 h travel	0.703 (0.699–0.707)	0.881 (0.878–0.883)	0.899 (0.898–0.901)
Private health facility <1 h travel	0.170 (0.167–0.173)	0.684 (0.681–0.688)	0.836 (0.834–0.839)
**Number of births/pregnancies‡**	54 250	61 285	100 990

*Variables constructed as averages at sub-district level.

†Variables constructed as averages at the sampled cluster level.

**‡**The number of births (for women who survived) or pregnancies (for women who died from maternal causes) is unweighted, means and 95% confidence intervals are based on weighted values.

In terms of the demographic characteristics of the sampled respondents, several notable changes occurred during the study period. Among all pregnancies, as fertility fell, the proportion of first pregnancies increased significantly across the survey rounds, with nearly 40% of all pregnancies being the first pregnancy in 2016. There was a mixed improvement in maternal risk profile: the proportion of births before age 20 increased significantly (*P* < 0.05), whereas the proportions of high parity births and births after 35 years of age declined significantly (*P* < 0.05). In terms of socioeconomic characteristics, the average years of education and the proportion of mothers living in the urban area increased significantly (*P* < 0.05) across all the survey rounds. The average household wealth index also increased significantly (*P* < 0.05) between 2001 and 2010 and between 2010 and 2016. All three maternal health services and access to public and private health facilities also increased significantly (*P* < 0.05) across all the survey rounds. Particularly the proportion of women within 1-hour of travel to the nearest private health facility increased from 17% in 2001 to 84% in 2016 (**Table 1**).

### Changes in the risk of maternal death between 2001–2010 and 2010–2016

The estimated risk ratios from Poisson regression models on the pooled data set to explore relations between the change in risk of maternal death are shown in **Table 2** [13,14]. Overall, the reduction in the risk of maternal death (**Table 2**) was significant during 2001–2010 (*P* < 0.001) but not during 2010–2016 (*P* = 0.066). Among demographic factors (**Table 2**), first pregnancy remained significantly protective against maternal death, but high parity births and births after 35 years significantly increased the risk of maternal death for both 2001–2010 and 2010–2016. Teenage pregnancy (i.e. pregnancy/birth before 20 years) was not significantly associated with the risk of maternal death during 2001–2010 (*P* = 0.338) but became significant during 2010–2016 by increasing the risk by more than 2-fold (*P* < 0.001).

**Table 2:** Associations of risk of maternal death and independent factors between 2010–2016*

Background characteristics	Model 1	Model 2	Model 3	Model 4	Model 5	Model 6	Model 7
**2001–2010**							
Survey round	0.630 (0.500–0.793)	0.717 (0.567–0.905)	0.703 (0.555–0.890)	0.659 (0.519–0.836)	0.775 (0.589–1.019)	0.670 (0.511–0.879)	0.827 (0.633–1.080)
First pregnancy		0.652 (0.453–0.938)					0.678 (0.468–0.981)
Birth parity ≥4		1.516 (1.154–1.992)					1.435 (1.081–1.904)
Birth at age <20		1.210 (0.819–1.789)					1.153 (0.778–1.709)
Birth at age ≥35		1.906 (1.420–2.559)					1.932 (1.438–2.598)
Years of education			0.945 (0.915–0.975)				1.000 (0.962–1.040)
Economic status				0.903 (0.831–0.980)			0.958 (0.871–1.054)
Urban residence				0.910 (0.684–1.211)			
≥4 ANC visits					1.401 (0.425–4.622)		
SBA					0.173 (0.031–0.962)		0.358 (0.133–0.967)
Cesarean sections					1.060 (0.060–18.66)		
Public facility <1 h						0.895 (0.680–1.179)	
Private facility <1 h						0.929 (0.700–1.233)	
Constant	0.003 (0.003–0.004)	0.003 (0.002–0.003)	0.004 (0.003–0.005)	0.005 (0.004–0.006)	0.004 (0.003–0.005)	0.004 (0.003–0.005)	0.003 (0.002–0.005)
**2010–2016**							
Survey round	0.804 (0.638–1.014)	0.964 (0.761–1.220)	0.926 (0.731–1.173)	0.798 (0.633–1.006)	0.943 (0.715–1.245)	0.813 (0.643–1.029)	0.965 (0.728–1.280)
First pregnancy		0.159 (0.100–0.254)					0.160 (0.100–0.257)
Birth parity ≥4		2.292 (1.762–2.982)					2.246 (1.698–2.970)
Birth at age <20		2.102 (1.429–3.092)					2.047 (1.389–3.019)
Birth at age ≥35		1.777 (1.301–2.427)					1.830 (1.337–2.505)
Years of education			0.917 (0.889–0.945)				1.014 (0.978–1.052)
Wealth status				0.863 (0.792–0.941)			0.917 (0.833–1.009)
Urban residence				0.814 (0.630–1.052)			
≥4 ANC visits					1.204 (0.516–2.810)		
SBA					0.230 (0.062–0.858)		0.899 (0.448–1.805)
Cesarean sections					2.237 (0.427–11.72)		
Public facility <1 h						0.746 (0.514–1.082)	
Private facility <1 h						0.967 (0.724–1.290)	
Constant	0.003 (0.002–0.004)	0.002 (0.001–0.003)	0.003 (0.002–0.005)	0.004 (0.003–0.007)	0.003 (0.002–0.004)	0.003 (0.002–0.006)	0.002 (0.001–0.004)

ANC – antenatal care, SBA – skilled birth attendants

*Estimates in the table are risk ratios with 95% confidence interval in parentheses.

Overall, one year increase in mothers’ education decreased the risk of maternal death by 5.5% during 2001–2010 and by 8.3% during 2010–2016 (**Table 2**), both of which were statistically significant (*P* < 0.001). Similarly, improvement in the household wealth index (quintiles) decreased the risk of maternal death by about 10% during 2001–2010 (*P* = 0.014) and by about 14% during 2010–2016 (*P* = 0.001). Living in urban areas was not significantly associated with the risk of maternal death during 2001–2010 or 2010–2016 (**Table 2**). Among the maternal care-seeking variables, only SBA remained significantly protective against maternal death throughout the study period. Both the subdistrict-level averages of having a cesarean section and making four or more antenatal care visits were associated with increased risk, though non-significantly (**Table 2**). None of the access to health services variables was found to be significantly associated with the risk of maternal death during 2001–2010 or 2010–2016 (**Table 2**).

After controlling for the covariates found to be significantly associated with the risk of maternal death from Models 1–6 in **Table** 2, only first pregnancies (*P* = 0.039), high parity births (*P* = 0.012), and pregnancy after 35 years (*P* < 0.001) remained statistically significant during 2001–2010. In contrast, the contributions of all the demographic factors were found to be strongly associated with the risk of maternal death during 2010–2016 (*P* < 0.001). Even after controlling for the first pregnancy and other relevant covariates, teenage pregnancy increased the risk of maternal death by 2.05 times (*P* < 0.001). After controlling for relevant covariates, contributions of maternal education and household wealth index lost their significance, though the proxy for socioeconomic status approached the significance level (*P* = 0.075) during 2010–2016. SBA was found to have statistically significant protection against maternal death during 2001–2010 (*P* = 0.043) but became not significant (*P* = 0.765) during 2010–2016 (**Table 2**).

## DISCUSSION

Our regression analyses showed a significant decline in the risk of maternal death during 2001–2010 (*P* < 0.001) but not during 2010–2016 (*P* = 0.066) (**Table 2**). This is in agreement with findings from the three rounds of BMMS, which estimated that the MMR in Bangladesh declined by 40% between 2001 and 2010 and remained unchanged between 2010 and 2016 [5]. Stalling of a decade-long decline in maternal mortality in Bangladesh is also supported by other data sources (Supplement 2 in the **Online Supplementary Document**). Such stalling of maternal mortality, despite improvements in delivery care coverage, is not uncommon (Supplement 3 in the **Online Supplementary Document**). Studies indicate that other countries have experienced stagnation in maternal mortality decline due to lower sociodemographic situation [2,15], poor sanitation [16], and inadequate care after delivery [17–19]. A “protracted” double burden of infectious and chronic diseases among women of reproductive age has also been found to be associated with stagnation in maternal mortality decline [19–21].

In our earlier analysis based on data from 2001 and 2010 rounds of BMMS, we identified factors both within and outside the health sector that contributed to Bangladesh’s decade-long maternal mortality decline [7]. Our analyses found that demographic factors continued to be significantly associated with maternal mortality. The first pregnancies remained protective, and high parity births and birth after age 35 increased the risk of maternal death significantly during both 2001–2010 and 2010–2016. While the maternal risk for high parity births has been well documented, the statistically significant protective effect of first pregnancy against maternal deaths was surprising since in the first pregnancy, compared to subsequent pregnancies, women are at higher risk of developing preeclampsia, a condition marked by hypertension and proteinuria, that may lead to maternal morbidity and mortality [22]. However, recent studies suggested that the increased risk of maternal mortality from hemorrhage or cardiovascular diseases for first births was not as great as previously believed [23]. Our analysis estimated the risk of maternal death among older-age mothers to be significantly high, which is in line with findings from other countries [24,25]. Bangladesh is already at an advanced stage of epidemiologic transition from communicable to non-communicable diseases, and the prevalence of hypertension and diabetes increased rapidly among women aged 35 years or over during the past decade [26,27], which may partly explain why the risk of maternal death did not decline among older mothers between 2001 and 2016.

A marked distinction between the risk factors of maternal death between 2001–2010 and 2010–2016 was the contribution of teenage (i.e. under 20 years) pregnancies. Even after controlling for the first pregnancy, teenage pregnancies significantly contributed to maternal death during 2010–2016 (**Table 2**). It indicated teenage pregnancies continued to be a significant risk factor for maternal death, irrespective of parity and the other factors that were controlled in the model. Since the first pregnancies continued to be significantly protective against maternal deaths, we did additional analyses to unpack the relationship between first pregnancy and pregnancies during teenage years in relation to maternal deaths (Supplement 4 in the **Online Supplementary Document**). After adding an interaction term between the first pregnancy and teenage pregnancy, we found that the first pregnancies among teenage mothers continued to be protective against maternal deaths (*P* = 0.001) (Table S1 in the **Online Supplementary Document**) but subsequent births among teenage mothers increased the risk of maternal deaths by nearly 3-fold between 2010 and 2016 (*P* < 0.001) (Table S1 in the **Online Supplementary Document**). Data from multiple rounds of the Bangladesh Demographic and Health Survey (BDHS) indicated that teenage childbearing has remained alarmingly high in Bangladesh, and most women completed their childbearing by their late twenties [28]. Between 1999 and 2017, a period roughly covering our study period, the prevalence of first births occurring to girls under 20 years decreased slowly in Bangladesh from 78 to 72%. However, the prevalence increased from 52 to 56% among girls aged 16–19 years during the same period [29]. Given that a third of all births will be to teenage mothers by 2025 in Bangladesh [30], multiple (i.e. more than one) pregnancies among young women will continue as a major contributor to maternal deaths in the coming years.

Among the health sector factors, SBA’s contribution significantly reduced the risk of maternal death during 2001–2010, but not during 2010–2016, after controlling for the relevant covariates. This finding was in line with other recent studies that demonstrated that increased coverage of critical maternal health services (e.g. facility deliveries) has not been adequate for reducing maternal or newborn deaths in developing countries [31–33]. Despite a near doubling of skilled deliveries between 2010 and 2016 (i.e. from 27 to 50%) (**Table 1**), the loss of SBA’s significance might also be explained by the persistently low level of readiness of health facilities for delivery services. Historically, the increase in SBA in Bangladesh has been primarily driven by facility delivery, which increased from 9% of total deliveries in 2001 to 23% in 2010, and to 47% in 2016 BMMS. A series of Bangladesh Health Facility Surveys (BHFS) between 2009 and 2017 provides nationally representative estimates of the readiness of public, NGO, and large (i.e. those with at least 20 beds) private hospitals to provide quality maternal health services. The BHFSs demonstrated that the readiness for maternal health services in public sector facilities was inadequate to provide quality delivery care. In 2017, 59% of facilities at district and sub-district levels had staff trained in delivery care; 23% had guidelines on basic or comprehensive emergency obstetric care (EmOC), and only 1% had all 13 tracer items necessary for normal delivery services [34].

Most of the increase in facility deliveries in Bangladesh happened in the private sector between 2010 and 2016 (from 48 to 62% of total facility deliveries) [5,9]. Thus, the service capacity at private health facilities is likely to be a strong factor influencing maternal deaths and morbidity. Private hospitals appear to be even less ready compared to the public sector sub-district and higher-level facilities to provide delivery care. Among the private hospitals providing normal deliveries, only 16% had staff trained in delivery care, 5% had guidelines on basic or comprehensive EmOC, and none had all 13 tracer items necessary for normal delivery services in 2017 [34]. Almost all private hospitals (with at least 20 beds) provided cesarean section delivery, but only 29% conducted all nine comprehensive EmOC signal functions [34]. There is no information on systems and service readiness for smaller than 20-bed private hospitals or clinics, where a substantial proportion of births may be occurring.

The two leading causes of maternal death are hemorrhage and eclampsia, accounting for 54% of maternal deaths in 2016 [35]. The readiness of the health system to address these and other maternal health complications is insufficient. In 2017, only 41% of all facilities that offer delivery services had staff trained in active management of the third stage of labor (AMTSL). Private hospitals were far less likely to have staff trained in AMTSL (15%) than the public sector sub-district and higher-level facilities (55%), although most deliveries occur in private facilities. Moreover, just 31% of health facilities that offer delivery services had supplies of injectable oxytocin to stop hemorrhage, and even fewer (14%) had injectable magnesium sulfate to treat eclampsia. Still, 53% of deliveries occur at home, mostly without skilled birth attendants, and community distribution of misoprostol for managing postpartum hemorrhage was only enough to cover about 17% of births in Bangladesh during 2015–2016 [36].

### Strengths and limitations

Among the low- or lower-middle-income countries, Bangladesh stands out as the only country that has conducted three comprehensive and reliable household sample surveys to measure maternal mortality at a national level. Our use of a rigorous multivariable regression model with random effects at the sub-district level enabled us to explore the relationship between the change in risk of maternal death from 2001 to 2016 and a range of demographic, socioeconomic, and health care factors. To our best knowledge, this is the first study that explores the association between the risk of maternal death and associated demographic, socioeconomic, and health care factors in Bangladesh using household-level data large enough to examine a rare event such as maternal mortality with acceptable precision.

Our analyses, however, have several limitations. As a result of constraints imposed by the survey data, we could not include any pregnancies that may have occurred during the three-year windows of observation to women who died of non-maternal causes. Pregnancies that did not result in a live birth (except for those pregnancies which resulted in a maternal death) were not included in our analyses as they fell outside the standard definition of maternal mortality. Like any retrospective survey, there is a possibility of recall bias during the data collection process of the BMMS. Additionally, it’s important to exercise caution when interpreting our proxy for respondents' socioeconomic status (i.e. household wealth index), as it is a relative index. Lastly, BMMS data did not collect information on the mother’s nutritional status, history of non-communicable diseases such as hypertension or diabetes, beliefs and social/cultural norms around delivery, and respectful maternity care, which could help us explain maternal health care seeking and risks of adverse delivery outcomes better. These should be considered as areas for further research.

## CONCLUSIONS

Bangladesh has made remarkable progress in increasing demands for and utilisation of maternal health services over the past two decades. While further research is needed to fully understand this stagnation of maternal mortality in Bangladesh, there is evidence of substantial deficiencies in the capacity and readiness to provide high-quality maternity care in both the public and private sectors. The findings of this study serve as a timely reminder that decreasing maternal mortality requires attention to both demand- and supply-side factors; maternal deaths will only be prevented if women avoid high-risk pregnancies and go to facilities for childbirth, and facilities are fully prepared to handle obstetric emergencies when they occur. Based on this study’s findings and evidence from other countries, we highlight the following policy and programmatic recommendations to reduce maternal mortality in Bangladesh. First, improve availability of FP services with an emphasis on spacing among young couples. Historically, Bangladesh’s FP initiatives have focused on reducing fertility levels and promoting clinical methods for limiting childbearing [37]. The government needs to take specific measures to reduce childbearing among teenage girls by promoting interval and postpartum contraception. Interventions to reduce adolescent fertility need to start before marriage to improve young girls’ knowledge of reproductive health [28]. Achieving this necessitates a comprehensive strengthening of Bangladesh's national FP programme and the improvement of FP service delivery under the ongoing sector-wide approaches for Bangladesh’s health sector. Second, increase ‘effective’ coverage of essential maternal health services through the public sector. Evidence indicates that increasing the public provisions of health care can accelerate the improvement of maternal health [38]. To improve the effective coverage of essential services, the government needs to: a) focus on key maternal health services with evidence to have a high impact in reducing maternal mortality, b) ensure an adequate supply of drugs and medical commodities, and c) build, allocate, and retain skilled providers at all levels. Third, improve readiness of health facilities to address major causes of maternal death. The availability of medicines to treat postpartum hemorrhage and eclampsia needs to be made universal at the health facilities and in the communities. Steps are needed to maintain a number of specific standards (e.g. availability of obstetricians and anesthesiologists for the comprehensive EmOC facilities) to ensure quality care at health facilities. Standard Operating Procedures (SOP) also need to be adopted and implemented at all levels of health facilities based on the National Strategy for Maternal Health 2019–2030 [39].

## Additional material


Online Supplementary Document
