# Supplementary material for: Stagnation of maternal mortality decline in Bangladesh between 2010 and 2016 in spite of an increase in health services utilisation: Examining data from three large cross-sectional surveys
Source: J Glob Health. 2024 Jan 26;14:04027. doi: 10.7189/jogh.14.04027 (PMC10811435; doi:10.7189/jogh.14.04027)
Supplement: Online Supplementary Document [file jogh-14-04027-s001.pdf]

SUPPLEMENTARY MATERIALS

Supplement 1. Notes on Maternal Mortality Ratio (MMR) estimation

Conventionally, the national maternal mortality ratios (MMRs) from BMMS data were obtained as follows: Maternal mortality rates (MMRates) for 2001, 2010, and 2016 surveys were calculated by dividing the number of maternal deaths in a particular age category by the exposure time (women-years) in that age category. The MMRs were then calculated by dividing each age-specific MMRate by the corresponding age-specific fertility rate, and expressed as the number of deaths per 100,000 live births. Standard errors for the MMRs were computed using the Jackknife repeated replications procedure.

For our statistical analysis, we decided to focus on obstetric risk, or risk per pregnancy, quantified by the MMR, as opposed to risk on a per-woman-year of exposure basis, represented by MMRate. This decision was influenced by the fact that the MMRate is heavily dependent on fertility levels, which is not our central focus; rather, our interest is centered on risk within each individual pregnancy. To that end, we pooled the dataset from birth records gathered in the three years leading up to each BMMS round, encompassing both surviving and deceased mothers. This data was obtained from the Bangladesh Maternal Mortality and Health Care Survey (BMMS) rounds conducted in 2001, 2010, and 2016. The included variables in our pooled dataset were diverse, encompassing whether the mother survived, whether any deaths were maternal, information on the mother's socioeconomic and demographic characteristics, and maternal health-seeking behaviors. It is important to note that for the years 2010 and 2016, healthcare utilization metrics were only obtainable for the sub-sample of mothers who were administered the more detailed version of the survey questionnaire.

Supplement 2: Comparison of the 2016 BMMS estimates of maternal mortality with other sources
All sample-based survey estimates are subject to sampling error, or uncertainty, since they are based on a sample of individuals rather than the whole population. Sampling errors relate to the fact that the chosen sample is only one of a very large number of samples which may have been chosen from the target population, each giving rise to different sample estimates. Using statistical theory, it is possible to say how precise a population estimate is by constructing a confidence interval (CI) around it to show the range of values which the true population value lies (i.e., the value that would have been found if the entire population had been surveyed). Following the standard practice for sample surveys, each round of BMMS estimated the 95% CI of MMR to assess change over time. MMR was estimated to be 196 maternal deaths with a 95% CI between 159 and 234, which means that MMR in Bangladesh was between 159 and 234 in the year 2015.

Figure S1. Maternal Mortality Ratio (MMR) estimates by BMMS

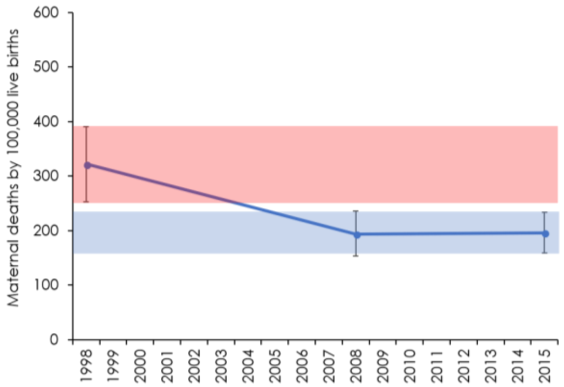

Figure S2. Comparison of 2015 MMR estimates from different sources

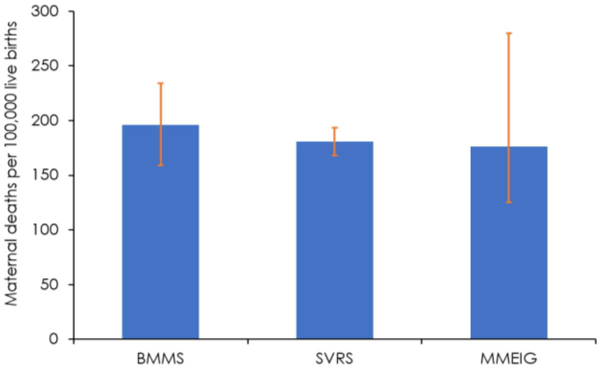

The 2016 BMMS demonstrated that there was no evidence of change in MMR In Bangladesh between 2008 and 2015—after a massive 40 percent reduction in MMR between 1998 and 2008 (mid-points of the reference period for MMR estimation in 2001 and 2010 BMMS rounds), MMR stayed at the same level between 2008 and 2015 with very similar Cis (blue-shaded area in Figure S1). As the Cis of the 2001 and 2010 BMMS estimates of MMR did not overlap (red- and blue-shaded areas in Figure S2), it can be concluded that there was a statistically significant decline in MMR between 1998 and 2008, and then plateaued between 2008 and 2015.

Comparison of MMR estimates by BMMS with other available sources (viz., Sample Vital Registration System [SVRS] of BBS and modeled estimate by the Maternal Mortality Estimation Inter-Agency Group [MMEIG]) demonstrates that though the point estimates vary from one source to another, the 95% Cis overlap (see Figure 2). This establishes that the MMR estimates for 2015 by SVRS, MMEIG and BMMS are similar, i.e., the difference between the estimates are not statistically significant. This is to be noted that the CI computed for MMEIG estimate refers to 80% uncertainty intervals (10th and 90th percentiles of the posterior distributions), which was chosen as opposed to the more standard 95% CI because of the substantial uncertainty inherent in maternal mortality outcomes.[1] As the 95% CI would be wider that the reported 80% CI, the overlapping of CIs would not have changed.

<u>Trends in MMR from Matlab Health and Demographic Surveillance System (HDSS)</u>
The International Centre for Diarrhoeal Disease Research, Bangladesh (icddr,b) has been maintaining a Health and Demographic Surveillance System (HDSS) in Matlab, a rural sub-district with 234,000 population located about 55 km southeast of Dhaka. The HDSS collects vital demographic and health information from each household in regular intervals. It also uses standardized Verbal Autopsy (VA) tools to collect information on cause of death for each deceased person in the surveillance area as well.[2]

In order to examine the trends in MMR in Matlab HDSS area, annual MMR was calculated for 15 years, from 2002 to 2016, based on around 5,000 live births every year. Due to small population size, it was expected that MMR would vary considerably from one year to another in Matlab HDSS (see Figure S3). However, linear trends for 2002–2010 and 2010–2016 indicate that decline in MMR stalled after 2010.

Figure S3. Trends in MMR from HDSS, 2002–2016

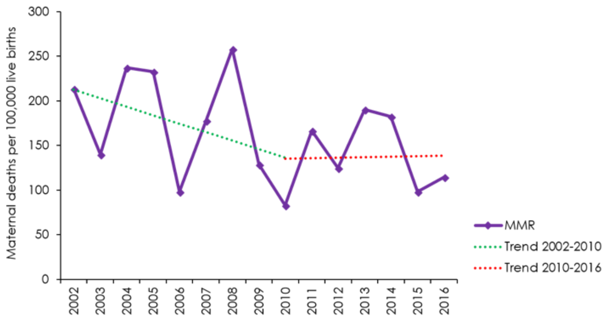

Figure S4. Trends in MMR from SVRS, 2002–2016

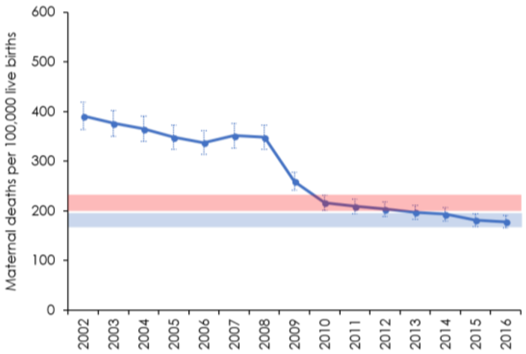

Trends in MMR from SVRS

Since 1980, BBS has been maintaining a Sample Vital Registration System (SVRS) to study the changes in the demographic events of Bangladesh during the intercensal periods. The coverage of SVRS increased from 103 primary sampling units (PSUs) in 1980, each consisting of 250 households, to 1000 PSUs in 2002 to 1500 PSUs in 2013. In 2015, an Integrated Multi-Purpose Sample (IMPS) design was followed to collect data on household and population characteristics, births, death, migration, marriage, disability, HIV/AIDS and contraceptive use from 2012 primary sampling units (PSUs).[3]

The 2015 SVRS reported that MMR has shown a consistent fall over the last five years, from 209 maternal deaths per 100,000 live births in 2011 to 181 in 2015. Since the CI of MMR was only reported for 2015 and 2016 estimates, simulated CIs (considering similar uncertainty levels of 2015/16) indicate that the 95% CIs of 2010 and 2015 MMR estimates are very close (red- and blue-shaded areas in Figure S4, respectively). Since the coverage of 2010 SVRS was 50 percent smaller than 2015 SVRS (i.e. 1,000 PSUs),[4] it is likely that the CI for 2010 would be wider and possibly overlap the CI for 2015, demonstrating a non-statistically significant MMR decline during 2010–2015. For 2015 and 2016, the estimated CI for MMR is very narrow (around 7 percent margin of error). Since the 2015 MMR estimate was based on around 17,675 births, the CI was supposed be wider. A review of SVRS estimate (including CI estimation) would be useful for tracking trends in MMR in the future.

Trends in MMR from MMEIG

In order to generate internationally comparable MMR estimates, the World Health Organization (WHO), the United Nations Children's Fund (UNICEF), the United Nations Population Fund (UNFPA), World Bank Group and the United Nations Population Division (UNPD) formed MMEIG. With independent advice from a technical advisory group that includes scientists and academics with experience in measuring maternal mortality, the MMEIG model generated estimates.

MMR estimates for 2015 were generated using a Bayesian approach, referred to as the Bmat model.[5,6] This model incorporates a number of covariates (i.e. gross domestic product per capita, general fertility rate, and coverage of skilled birth attendants), but prioritize country-level data on maternal mortality when available, to estimate MMR.[10] As the modeled MMEIG estimates have a very wide CI, it shows that there was no statistically significant reduction in MMR between 2003 and 2015 (blue-shaded area in Figure S5). If the Cis were 95% instead of 80% as calculated by MMEIG, it would indicate that MMR decline during 2000–2015 was not significant.

Figure S5. Trends in MMR from MMEIG, 1998–2015

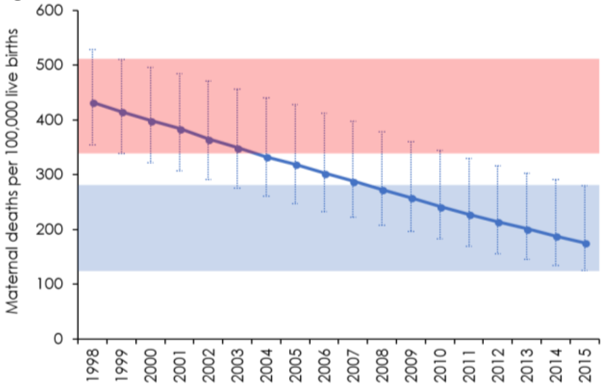

Figure S6. Maternal deaths in 2015 from HIS

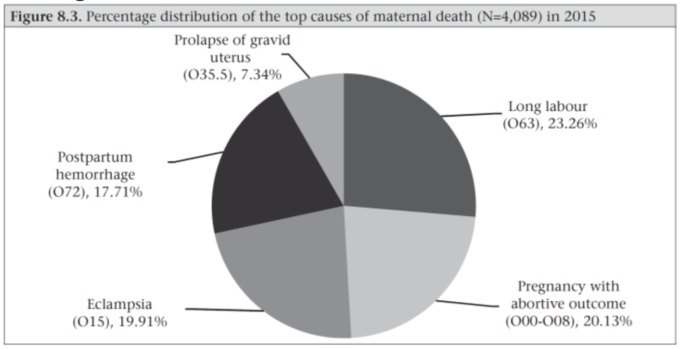

Maternal mortality data from Health Information System (HIS)

Valuable information can be obtained when maternal deaths that occur in a health facility are reviewed specifically to identify where the health system needs to improve. However, in low- and middle-income countries unless more than 95 percent of women give birth in a health facility (as opposed to at home), hospital-based data cannot be used to provide accurate estimates of MMR for the population.[6] In order to compare maternal mortality information from the HIS of the Directorate General of Health Services with the 2016 BMMS, estimated total number of maternal deaths in a year was considered. Based on hospital and health-facility based information, the HIS reported that 4,089 maternal deaths took place in 2015 (see Figure S6).[7] The 2016 BMMS estimated that about two-thirds of the maternal deaths take place in health facilities (i.e., facilities in public, private and NGO sectors). Using the HIS data for 2015 for maternal deaths in public facilities (and a number of private and NGO hospitals which provide emergency obstetric care[8]), the total number of maternal deaths in 2015 becomes 4089÷0.66=6,195 deaths. This figure is very close to the 2016 BMMS estimated total number of maternal deaths in a year (6,076).

While comparing the 2016 BMMS estimate of MMR with other sources like Matlab HDSS, SVRS and MMEIG, it can be observed that: a) the estimated MMRs from different sources are not significantly different and therefore indicate an equivalent level for 2015; and b) plateauing of MMR level during 2010–2015 as reported in the 2016 BMMS can be observed in other sources as well. Based on this assessment, the 2016 BMMS estimates are found to be in agreement with other sources.

Figure S7. MMR stalling at high, medium or low levels in selected countries[9]

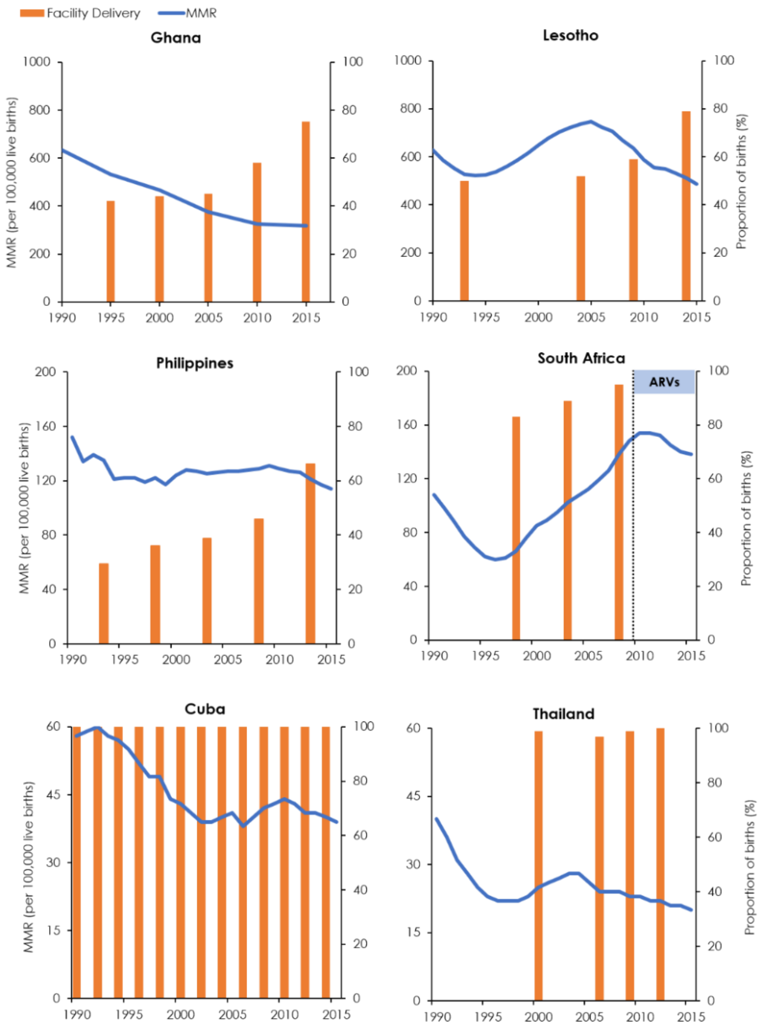
Facility Delivery
MMR
Ghana
Lesotho
Philippines
South Africa
ARVs
Cuba
Thailand
MMR (per 100,000 live births)
Proportion of births (%)
1990
1995
2000
2005
2010
2015

Supplement 3: Stalling of MMR decline in other countries
Is no reduction of MMR between 2010 and 2016 observed in Bangladesh, particularly when maternal healthcare utilization has continued to increase, exceptional? The answer is no, as there is international precedence for a stall in MMR decline after a secular trend. MMR has stalled in 37 countries in the world despite improvement in many aspects of healthcare, the association was particularly weak in the nine South and Southeast Asian countries, most of which had an MMR of around 200 per 100,000 live births.10 In some of these countries the apparent stall in MMR occurred despite increases in coverage of skilled birth attendance and health facility deliveries. Bangladesh is therefore not the only country to experience this pattern. Figure S7 illustrates stalling or fluctuating MMR in selected countries and Figure S8 suggests that a high level of facility delivery is important but not sufficient to lower MMR across countries.

Figure S8. MMR and facility deliveries in 37 Sub-Saharan African and South/Southeast Asian countries[11]

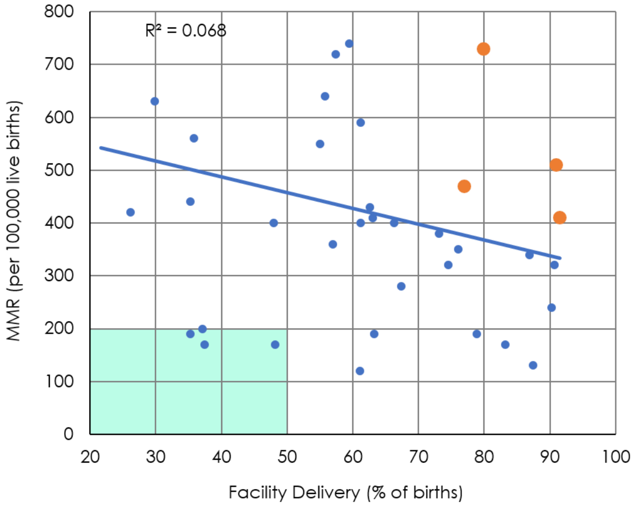

Supplement 4. Additional regression analysis

Since the first pregnancies continued to be significantly protective against maternal deaths during 2001, we did additional analyses to unpack the relationship between first pregnancy and pregnancies during teenage years in relation to maternal deaths. After adding an interaction term between the first pregnancy and teenage pregnancy in Model 7, we found that the first pregnancies among teenage mothers continued to be protective against maternal deaths (p=0.001) but subsequent births among teenage mothers increased the risk of maternal deaths by nearly three-fold between 2010 and 2016 (p<0.001).

Table S1. Additional analysis on the associations of risk of maternal death and independent factors between 2001–2010 and 2010–2016

**Background characteristics**	**Survey rounds**	
	2001–2010	***2010–2016***
Survey round	0.830 (0.635-1.086)	0.972 (0.733-1.289)
First pregnancy	0.806 (0.529-1.226)	0.323 (0.188-0.555)
Birth parity ≥4	1.473 (1.106-1.962)	2.330 (1.758-3.088)
Birth at age <20	1.500 (0.909-2.475)	2.785 (1.882-4.123)
Birth at age ≥35	1.946 (1.447-2.618)	1.858 (1.356-2.546)
First pregnancy x Birth at age <20	0.573 (0.272-1.206)	0.233 (0.098-0.558)
Years of education	0.999 (0.961-1.039)	1.012 (0.976-1.050)
Economic status	0.958 (0.871-1.054)	0.916 (0.832-1.009)
SBA	0.358 (0.132-0.967)	0.906 (0.451-1.819)
Constant	0.003 (0.002-0.004)	0.002 (0.001-0.004)
N	115,534	162,275

Notes: Estimates in the table are risk ratios with 95% confidence interval in parentheses.

[1] World Health Organization (WHO). Trends in maternal mortality 1990 to 2015: estimates by WHO, UNICEF, UNFPA, World Bank Group and the United Nations Population Division. Geneva: WHO; 2015.

[2] Icddr,b. Health and Demographic Surveillance System–Matlab, Volume 50. Registration of health and demographic events 2015, Scientific Report No. 135. Dhaka: icddr,b; 2017.

[3] Bangladesh Bureau of Statistics (BBS). Report on Bangladesh Sample Vital Statistics 2015. Dhaka: BBS; 2016.

[4] Bangladesh Bureau of Statistics (BBS). Report on Bangladesh Sample Vital Statistics 2010. Dhaka: BBS; 2011.

[5] Alkema L, Chou D, Hogan D, Zhang S, Moller AB, Gemmill A, Fat DM, Boerma T, Temmerman M, Mathers C, Say L. Global, regional, and national levels and trends in maternal mortality between 1990 and 2015, with scenario-based projections to 2030: a systematic analysis by the UN Maternal Mortality Estimation Inter-Agency Group. *The Lancet*. 2016; 387(10017):462-74.

[6] Alkema L, Zhang S, Chou D, Gemmill A, Moller AB, Fat DM, Say L, Mathers C, Hogan D. A Bayesian approach to the global estimation of maternal mortality. *The Annals of Applied Statistics*. 2017; 11(3):1245-74.

[7] Management Information System. Health Bulletin 2016. Dhaka: Directorate General of Health Services; 2017.

[8] Management Information System. Voice of MIS-Health: Emergency Obstetric Care (EmOC) Performance Report 2016. Newsletter Issue No. 14. Dhaka: Directorate General of Health Services; 2017.

[9] Joint UNICEF/WHO database of skilled health personnel, based on population based national household survey data and routine health systems. 2016. Available from: https://data.unicef.org/topic/maternal-health/delivery-care/

[10] World Health Organization (WHO). Trends in maternal mortality: 1990 to 2015: estimates by WHO, UNICEF, UNFPA, World Bank Group and the United Nations Population Division. Geneva: WHO; 2015.

[11] Maternal Mortality Estimation Inter-Agency Group (MMEIG). (2015). Trends in maternal mortality 1990 to 2015: Estimates by WHO, UNICEF, UNFPA, World Bank Group, and the United Nations Population Division. Geneva, Switzerland: World Health Organization.
